# Eight Million Years of Satellite DNA Evolution in Grasshoppers of the Genus *Schistocerca* Illuminate the Ins and Outs of the Library Hypothesis

**DOI:** 10.1093/gbe/evaa018

**Published:** 2020-03-17

**Authors:** Octavio M Palacios-Gimenez, Diogo Milani, Hojun Song, Dardo A Marti, Maria D López-León, Francisco J Ruiz-Ruano, Juan Pedro M Camacho, Diogo C Cabral-de-Mello

**Affiliations:** e1 Department of Evolutionary Biology, Evolutionary Biology Centre, Uppsala University, Sweden; e2 Departamento de Biologia Geral e Aplicada, Instituto de Biociências/IB, UNESP – Univ Estadual Paulista, Rio Claro, São Paulo, Brazil; e3 Department of Entomology, Texas A&M University; e4 Laboratorio de Genética Evolutiva, IBS, Facultad de Ciencias Exactas, Químicas y Naturales, Universidad Nacional de Misiones, CONICET, Posadas, Argentina; e5 Departamento de Genética, Facultad de Ciencias, UGR – Univ de Granada, Spain; e6 Department of Organismal Biology, Systematic Biology, Evolutionary Biology Centre, Uppsala University, Uppsala, Sweden

**Keywords:** chromosomal evolution, genome organization, tandem repeats, repetitive DNA

## Abstract

Satellite DNA (satDNA) is an abundant class of tandemly repeated noncoding sequences, showing high rate of change in sequence, abundance, and physical location. However, the mechanisms promoting these changes are still controversial. The library model was put forward to explain the conservation of some satDNAs for long periods, predicting that related species share a common collection of satDNAs, which mostly experience quantitative changes. Here, we tested the library model by analyzing three satDNAs in ten species of *Schistocerca* grasshoppers. This group represents a valuable material because it diversified during the last 7.9 Myr across the American continent from the African desert locust (*Schistocerca gregaria*), and this thus illuminates the direction of evolutionary changes. By combining bioinformatic and cytogenetic, we tested whether these three satDNA families found in *S. gregaria* are also present in nine American species, and whether differential gains and/or losses have occurred in the lineages. We found that the three satDNAs are present in all species but display remarkable interspecies differences in their abundance and sequences while being highly consistent with genus phylogeny. The number of chromosomal loci where satDNA is present was also consistent with phylogeny for two satDNA families but not for the other. Our results suggest eminently chance events for satDNA evolution. Several evolutionary trends clearly imply either massive amplifications or contractions, thus closely fitting the library model prediction that changes are mostly quantitative. Finally, we found that satDNA amplifications or contractions may influence the evolution of monomer consensus sequences and by chance playing a major role in driftlike dynamics.

## Introduction

Satellite DNA (satDNA) is a heterogeneous collection of noncoding nuclear sequences that exists in hundreds to thousands of copies tandemly arranged at one or more chromosomal loci. Across eukaryotes, it is often located within the gene-poor heterochromatin of centromeres and telomeres, albeit satDNAs have also been reported on euchromatin in some species (see Charlesworth et al. 1994; Ugarković and Plohl 2002; Larracuente 2014; Ruiz-Ruano et al. 2016; Garrido-Ramos 2017). SatDNA generally shows a concerted pattern of evolution implying higher similarity for repeats within species than between species at intra- and inter-chromosomal levels (Dover 1982; Ugarković and Plohl 2002; Plohl et al. 2008; Garrido-Ramos 2017). Unequal crossing over, intrastrand homologous recombination, gene conversion, slippage rolling-circle replication, and transposition are the main factors driving the gradual turnover of satDNA variants produced by mutation, within and between loci, causing homogenization (Smith 1976; Dover 1982, 2002; Walsh 1987; Shi et al. 2010). Out of these mechanisms, only those changing copy number (e.g., unequal crossing over) can yield changes in satDNA abundance, either gains (amplification) or losses (contraction) (Lower et al. 2018).

The high sequence divergence observed for satDNA at interspecific level is a consequence of relaxed evolutionary constraints, in most cases due to lack of apparent function, so that changes in copy number and sequence variants are likely neutral (Lower et al. 2018). Therefore, satDNA sequences change so fast that they fade away after long evolutionary times. For instance, Ruiz-Ruano et al. (2016, 2017, 2018) reported the satellitomes in three grasshopper species belonging to three orthopteran families (Pyrgomorphidae, Pamphagidae, and Acrididae) dating back about 140, 111 and 73 Ma, respectively (Song et al. 2015), and none of the 165 satDNA families found showed interspecific homology, apart from the telomeric repeat. However, at shorter evolutionary scale, it is still possible to find homologous satDNAs between species. There are many cases of satDNA homology between species along short periods. This is the case, for instance, of baleen whales (Arnason et al. 1992), the *Drosophila obscura* group (Bachmann and Sperlich 1993), or the *Peromyscus* genus (Smalec et al. 2019), all comprising evolutionary periods shorter than 5 Myr. However, there are also exceptional cases of extremely long duration, such as, for instance, the 15–20 Myr in Bovidae (Escudeiro et al. 2019), 74–80 Myr in ants (Lorite et al. 2017), or 540 Myr in moluscs (Plohl et al. 2010).

The library model (Salser et al. 1976; Fry and Salser 1977) states that related species share a collection of satDNA sequences that may contract or expand, in one or another species, during evolution. After speciation events, independent reproduction is expected to force the evolution of species-specific profiles of satDNA sequence variants (Ferree and Barbash 2009; Ferree and Prasad 2012; Gallach 2014). The finding of homologous satDNA at such a variety of evolutionary times makes it very complex to visualize the satDNA library beyond some partial glimpses by analyzing one or more satDNAs on groups of species with higher or lower relativeness (e.g., Mestrović et al. 1998; Silva et al. 2017; Utsunomia et al. 2017; Šatović and Plohl 2018; Smalec et al. 2019).

The genus *Schistocerca* (Orthoptera, Acrididae, Cyrtacanthacridinae) includes some of the most damaging swarming locust species, including the African desert locust (*S. gregaria*), the Central American locust (*S. piceifrons*), and the South American locust (*S. cancellata*) (Harvey 1981; Song et al. 2017). These species exhibit an extreme form of density-dependent phenotypic plasticity, commonly referred to as locust phase polyphenism in which cryptically colored, shy individuals can transform into conspicuously colored, gregarious individuals in response to increases in population density (Uvarov 1966; Pener 1983; Simpson and Sword 2009). Most of what we know about locust phase polyphenism comes from decades of research on *S. gregaria* (Pener and Simpson 2009; Cullen et al. 2017). The genus *Schistocerca* includes about 50 species, most of which are nonswarming sedentary species, and thus the genus has been considered an excellent system to study the evolution of phenotypic plasticity (Song and Wenzel 2008; Song et al. 2017). The genus also shows a peculiar biogeographical distribution in which only the desert locust *S. gregaria* is found in the Old World (Africa and the Middle East), whereas the other species are distributed throughout America (Lovejoy et al. 2006; Song et al. 2013, 2017). Recent phylogenetic hypotheses about *Schistocerca* evolution, based on molecular data (Lovejoy et al. 2006; Song et al. 2013, 2017), have consistently shown that the desert locust is the earliest diverging lineage within the genus, supporting the hypothesis of an Old World origin for the genus (Kevan 1989; Ritchie and Pedgley 1989). It has been hypothesized that the ancestral *Schistocerca* colonized the New World through westward transatlantic flight about 6 Ma (Song et al. 2017), where it radiated and gave rise to the current diversity of species in the New World. Although the evolution of phenotypic plasticity in *Schistocerca* is an active area of research (Cullen et al. 2017), little is known about genome and chromosome evolution in this genus, and currently there is no reference genome sequenced for any *Schistocerca* species. So far, chromosomal information is limited to six species which display the typical karyotype for Acridid grasshoppers, with 2*n* = 22 + X0♂/XX♀ acro-telocentric chromosomes (White 1934; Mesa et al. 1982; Souza and Mello 2007; Milani et al. 2018). In addition, chromosomal mapping of repetitive DNA is even more limited, as only a few sequences in three species are currently known (Souza and Mello 2007; Camacho et al. 2015; Milani et al. 2018). Therefore, there is a clear need to study the molecular composition of chromosomes in *Schistocerca*, which could be useful for understanding genome organization and evolution in the genus.

Grasshopper genomes are very large (Gregory 2019) and contain high amounts of repetitive DNA (Wang et al. 2014), which makes them a good model for analysis of satDNAs evolution. Here, we analyze the SG1, SG2, and SG3 satDNA families in *S. gregaria* and in nine American species to find out if they have experienced changes in copy number and sequence during species radiation in America, by combining high-throughput sequencing, bioinformatic mining, and fluorescence in situ hybridization (FISH) mapping. The knowledge that *S. gregaria* is basal in the *Schistocerca* phylogeny (see above) constitutes a privileged situation to analyze evolutionary changes in the satDNA library since these species diversification, 7.9 Ma, as it allows inferring change direction. We found that the three satDNA families are still present in all species, with high interspecific differences in abundance, sequence, and chromosome location, which are explained by massive gains and/or losses of satDNA in some species. This confirms that satDNA mainly evolves through quantitative changes in abundance due to local amplifications or contractions. In addition, sequence evolution of satDNA closely resembles the species phylogeny, with higher similarity between closely related species, a fact also predicted by the library model.

## Materials and Methods

### Species Sampling, Chromosome Spreading, C-Banding, and DNA Extraction

Male and female adult grasshoppers were collected at the following localities: *Schistocerca serialis cubense* (SSEC), *S. americana* (SAME), *S. damnifica* (SDAM), *S. ceratiola* (SCER), and *S. rubiginosa* (SRUB) (Florida, USA), *S. caribbeana* (SCAR) (St. John, US Virgin Islands), *S. pallens* (SPAL) (Ñu Pyahú, Argentina), *S. cancellata* (SCAN) (Caucete, Argentina), and *S. flavofasciata* (SFLA) (Rio Claro, São Paulo, Brazil). *Shistocerca gregaria* (SGRE) information was obtained from Camacho et al. (2015). To visualize meiotic chromosomes, testes were dissected and fixed in modified Carnoy’s solution (3:1, 100% ethanol:glacial acetic acid). For mitotic chromosomes, female gastric ceca were removed and processed as described elsewhere (Castillo et al. 2011). All animal bodies were stored in absolute ethanol until DNA extraction by the traditional phenol:chloroform method (Sambrook and Russel 2001). We analyzed general chromosome features using Giemsa stained (5%) preparations. C-banding was performed according to Sumner (1972).

### Illumina Sequencing and Computational Analysis of SG1, SG2, and SG3 satDNAs in *Schistocerca*

We used the genomic DNA sequencing reads from *Schistocerca* species previously generated by Song et al. (2017). Paired-end Illumina reads were preprocessed to check quality by FASTQC ([Bibr evaa018-B1][Bibr evaa018-B1]010) and they were quality-trimmed using Trim Galore (https://github.com/FelixKrueger/TrimGalore/, last accessed March 18, 2019). The trimmed paired-end reads were joined by using the “fastq-join” software of the FASTX-Toolkit suit (Gordon and Hannon 2010), with default options.

We used three approaches to search for satDNA sequences across *Schistocerca* species. First, we performed graph-based clustering and de novo assembly of these sequences using the RepeatExplorer pipeline (Novák et al. 2010, 2013), setting default options using as input 500,00 reads. Then, we used BlastN to search for satDNAs in every final RepeatExplorer contig output, using the SGRE SG1 (KJ649466), SG2 (HG965751–HG965758), and SG3 (KJ649467) satDNA consensus sequences as query. Second, if no BlastN hit was retrieved by means of the previous methodology, we applied NOVOPlasty (https://github.com/ndierckx/NOVOPlasty/, last accessed April 13, 2019) to perform de novo assembling using SG1, SG2, and SG3 sequences as seed input and k-mer 21–23 as parameters. This second strategy was used for SG1 of SDAM for SG2 of SSEC, SPAL, SRUB, SDAM, SFLA, and SCAR. Third, we used RepeatMasker (Smit et al. 2013–2015) to map the Illumina reads on the consensus SG1–SG3 sequences, in order to estimate the abundance and divergence of each satDNA in each species. We then expressed abundance as the proportion of nucleotides mapped with the reference sequence in respect to total library size. All three reference sequences consisted in a dimer of the satDNA consensus sequence. We used 4 million random raw read pairs (2 million of each library), obtained by the seqtk tool (https://github.com/lh3/seqtk/, last accessed April 13, 2019), to estimate satDNA abundance and divergence with RepeatMasker. To determine the fraction of reads showing similarity with a given target satDNA, we used -s -inv -a -nolow -no_is -e rmblast and a custom library containing the three satDNA sequences, as parameters in RepeatMasker. Using the generated files, we estimated the Kimura 2-parameter (K2P) distances of each satDNA fragment against the reference consensus sequence, using the calcDivergenceFromAlign.pl script from the RepeatMasker utilities. A so-called satDNA landscape that depicts the relative abundance of repeat elements on the *y* axis and the K2P distance (i.e., satDNA divergence) on the *x* axis was made to visualize the changes in abundance for satDNA variants showing different degree of divergence. Finally, the reads mapped for every satDNA, by RepeatMasker, were collected and assembled using the CAP3 assembly software (Huang and Madan 1999).

The contigs identified as the three target satDNAs (i.e., SG1, SG2, and SG3), by our three assembly approaches, were submitted to the dotplot graphic alignment tool implemented in Dotlet (Junier and Pagni 2000) to identify the exact start and end of tandem monomer repeats, and to the Tandem Repeat Finder (TRF) algorithm (Benson 1999) to identify sequences with maximized alignment scores between the different tandemly repeated monomers. Tandem Repeat Finder alignment parameters used were 2, 3, and 5 for match, mismatch, and indels, respectively, and a minimum alignment score of 50.

We used Muscle (Edgar 2004), implemented in the MEGA5 software (Tamura et al. 2011), to perform multiple sequence intra/interspecific alignments, and to estimate A + T content and repeat lengths of the monomer’s copy recovered in the assembly. Phylogenetic reconstruction of the satDNA sequences was inferred by maximum likelihood (ML) trees using the K2P model distance implemented in MEGA5 (Tamura et al. 2011). To examine satDNA polymorphism within and between species, the aligned copies were subjected to an analysis using DnaSP v.5.10.01 (Librado and Rozas 2009) to compute the basic sequence statistics. Satellite consensus sequences have been deposited to GenBank under the MK948933–MK948959 access numbers. Satellite alignments are available upon request to the authors.

To analyze satDNA changes in these species, in an evolutionary context, we traced satDNA abundance on a molecular phylogeny previously built by Song et al. (2017). It covers almost 8 Myr of evolutionary history for the genus *Schistocerca* and includes all ten species analyzed here (supplementary fig. 1, Supplementary Material online), with SGRE being the ancestor of all American species, and the nine remaining species analyzed here placed at four different clades. Clade 1 included SFLA and SCAR, clade 2 included SPAL and SCAN, clade 3 included SSEC and SAME along with other species not analyzed here, and clade 4 included SDAM, SCER, and SRUB. Whereas clades 1 and 2 each comprised pairs of sister species, and clades 3 and 4 were more complex by also including some species not analyzed here.

To find out possible tendency directions in satDNA change among species, we calculated *z*-score standardized values for abundance (zs-abun) and divergence (zs-div) as the difference between the species value and the mean for all species, divided by the standard deviation of the former mean. This allows expressing changes as gains or losses, in respect to the average values for these ten species.

To analyze changes in DNA sequence for SG1–SG3 during the 7.9 Myr radiation of the genus *Schistocerca*, we used the consensus sequence for the monomer of each satDNA in each species and then calculated pairwise distance (*D*) by means of *p* distance in MEGA5, which expresses the proportion of differing nucleotide sites. As molecular phylogenetic analysis indicated that American species derived from a common radiation event in which SGRE is basal, we finally got an estimate of substitution rate for each satDNA in each species by dividing the *p* distance in respect to SGRE by twice the time since the radiation (15.8 Myr).

### Polymerase Chain Reaction Amplification of satDNAs and FISH

We used polymerase chain reaction (PCR) to amplify each satDNA in each species and to test its presence and generate DNA probes for FISH. We performed PCR using the SG1 and SG2 primers described in Camacho et al. (2015). The SG3 sequences showed higher variation among species, for which reason we used the assembled sequences to design four sets of primers: set A (F: 5′GAGGGATGCAGTAAAGAAGG, R: 5′GCTCCCTCACATTGACGAAT), set B (F: 5′AGCGTTGCAGTAAGGAACGA, R: 5′CGCTCCCTCACATTGACGA), set C (F: 5′GCGAGGGATGCACTAAAGAA, R: 5′TCCCGCACACTGACGAATTA), and set D (F: 5′-CGCAGGATACAGTAAAGAAG, R: CACCCTCACATTGACGATTT). We used the A set primers for PCR reactions in SCER, SDAM, SRUB, and SSEC, the B set in SAME, the C set in SCAN and SPAL, and the D set in SFLA and SCAR. PCR was performed using 10× PCR Rxn Buffer, 0.2 mM MgCl_2_, 0.16 mM dNTPs, 2 mM each primer, 1 U of Platinum *Taq* DNA Polymerase (Invitrogen, San Diego, CA) and 50–100 ng/μl template DNA. The conditions were as follows: initial denaturation at 94 °C for 5 min and 30 cycles at 94 °C (30 s), 55 °C (30 s), and 72 °C (80 s), plus a final extension at 72 °C for 5 min. The obtained fragments were separated by electrophoresis on 1% agarose gel. The monomer bands were cut out of the gel and purified using the Zymoclean Gel DNA Recovery Kit (Zymo Research Corp., The Epigenetics Company, USA) according to manufacturer’s recommendations. These products were used for reamplification through PCR using the same conditions mentioned above. Monomers were purified using ExoSAP-IT PCR product cleanup reagent (Thermo Fischer) and sequenced by the Sanger method in both directions using the Macrogen Inc. service (Korea) to confirm the identity of the desired sequences.

We performed single FISH mapping to detect the chromosomal location of satDNAs, according to the protocol published elsewhere (Cabral-de-Mello et al. 2010), and using the satDNA monomers obtained for each species as probes. FISH data for SGRE were obtained from Camacho et al. (2015). Probes were labeled through nick translation using biotin-14-dATP (Invitrogen) or digoxigenin-11-dUTP (Roche, Mannheim, Germany). The probes labeled with digoxigenin-11-dUTP were detected using anti-digoxigenin-rhodamine (Roche), whereas the probes labeled with biotin-14-dATP were detected using Streptavidin, Alexa Fluor 488-conjugated (Invitrogen). Preparations were counterstained using 4′,6-diamidine-2′-phenylindole and mounted in VECTASHIELD (Vector, Burlingame, CA). The chromosomes and hybridization signals were observed using an Olympus microscope BX61 equipped with a fluorescent lamp and appropriate filters. Fluorescent images were recorded using a DP71 cooled digital camera in gray scale. The images were pseudocolored in blue (chromosomes) and red (hybridization signals), merged, and optimized for brightness and contrast using Adobe Photoshop CS6. We classified the FISH signals on the chromosomes as proximal, interstitial, or distal to the centromere.

## Results

### Karyotypes and Heterochromatin

**Fig. 1.**
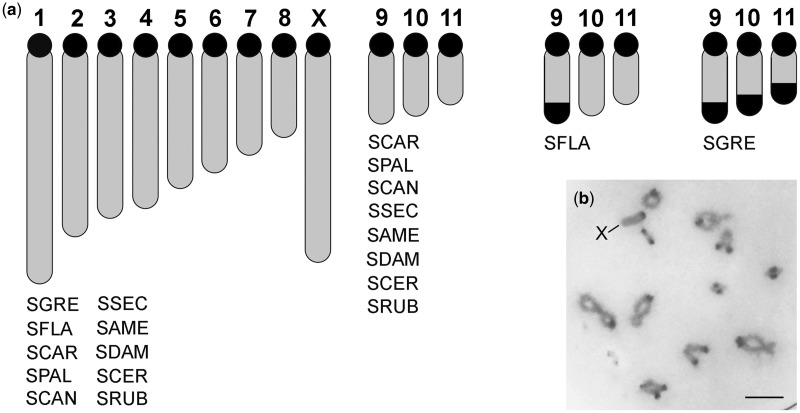
—C-banding revealing the heterochromatin location in *Schistocerca* species. (*a*) Ideogram showing heterochromatin distribution (C-positive blocks) in the haploid chromosome complement of *Schistocerca* species. The black dots are the centromeric heterochromatin, and the black distal block in chromosome 9 of SFLA as well as chromosome 9–11 of SGRE corresponds to the extra C-positive bands. (*b*) Representative C-banding in Diplotene chromosome spreads from SAME showing the pericentromeric location (most common location) of C-positive block (dark band) in *Schistocerca*. Bar in (*b*) corresponds to 5 μm.

All ten species studied here showed the typical karyotype reported for *Schistocerca* and Acrididae grasshoppers (see the Introduction section). Constitutive heterochromatin (revealed by the presence of dark bands on chromosomes after C-banding) was present on pericentromeric regions of all chromosomes in all species (fig. 1 and supplementary fig. 2, Supplementary Material online) and also on terminal regions of autosome 9 in SFLA (in consistency with previous reports by Souza and Mello 2007) and autosomes 9–11 in SGRE (Camacho et al. 2015). In SCER (supplementary fig. 2, Supplementary Material online) and SRUB, all C-bands were smaller than those in the remaining species (see Milani et al. 2018). Finally, SRUB carried an additional B chromosome showing a large pericentromeric heterochromatin block (Milani et al. 2018).

### Satellite DNA Detection and Sequence Analysis

Our bioinformatic analyses by means of RepeatExplorer, BlastN, NOVOPlasty, and RepeatMasker revealed the presence of the SG1, SG2 and SG3 satDNA families in the nine American *Schistocerca* species. Consistently, PCR amplification of the three satDNAs on genomic DNA showed, in all nine species, the ladder-type patterns typical for satDNAs, but they were not amplified in AAGE. The monomeric satDNA bands were extracted from the gel, sequenced, and aligned to the SGRE satDNA sequences, and this confirmed their homology. The main features observed for the SG1, SG2, and SG3 satDNA families in the ten *Schistocerca* species are summarized in table 1. The two sequence-dependent characteristics of these satDNAs (i.e., monomer size and A + T) showed very scarce variation among species (CV < 6% in all cases). In contrast, SG1 showed higher average abundance and divergence than SG2 and SG3, but these two features showed extensive variation among species (CV between 33.2% and 193.7%), although SG2 was the satDNA showing the highest CV (193.7% for abundance and 52.7% for intraspecific K2P divergence). Remarkably, the latter parameter was, on average, lower than interspecific K2P for all three satDNAs (see table 1), as expected from concerted evolution.

**Table 1:** Main Features of SG1, SG2, and SG3 satDNAs Locus Repeat Families in *Schistocerca* Species Analyzed by DNA-seq Data

Repeat Family	Species	Monomer Size (bp)	A + T%	Abundance (% Genome)	Copy Number	Intraspecific Kimura Divergence (%)	Interspecific Kimura Divergence (%)
SG1	*S. gregaria* - SGRE	171	55	6.7	161,077	8.97	
	*S. flavofasciata* - SFLA	178	56.7	1.5	72,503	13.25	
	*S. caribbeana* - SCAR	175	56.6	1.6	78,458	14.57	
	*S. pallens* - SPAL	173	61.3	2.84	133,314	17.47	
	*S. cancellata* - SCAN	170	51.2	1.74	96,748	23.55	
	*S. serialis cubense* - SSEC	170	58.8	0.47	21,338	14.96	
	*S. americana* - SAME	170	60	0.55	2,540	10.66	
	*S. damnifica* - SDAM	170	56.3	0.47	29,448	27.29	
	*S. ceratiola* - SCER	170	60	0.07	3,624	27.93	
	*S. rubiginosa -* SRUB	171	60.2	0.1	7,761	19.54	
	Average	172	57.6	1.6	60,681	17.8	27.5
	SD	3	3.1	2.0	56,834	6.6	
	CV (%)	1.6	5.3	124.4	93.7	37.3	
SG2	*S. gregaria* - SGRE	350	57	4.17	94,563	6.66	
	*S. flavofasciata* - SFLA	352	55.7	0.38	17,282	6.19	
	*S. caribbeana* - SCAR	352	55.5	0.27	12,210	8.92	
	*S. pallens* - SPAL	352	57.1	0.09	4,093	24.1	
	*S. cancellata* - SCAN	352	57.1	0.3	13,667	20.82	
	*S. serialis cubense* - SSEC	342	59.6	0.07	3,219	10.7	
	*S. americana* - SAME	336	62.2	0.01	62	21.92	
	*S. damnifica* - SDAM	341	59.8	0.01	248	12.65	
	*S. ceratiola* - SCER	353	56.4	0.03	748	13.61	
	*S. rubiginosa -* SRUB	343	58.6	1.35	59,827	5.49	
	Average	347	57.9	0.7	20,592	13.1	26.4
	SD	6	2.1	1.3	31,523	6.9	
	CV (%)	1.8	3.7	193.7	153.1	52.7	
SG3	*S. gregaria* - SGRE	170	52.9	0.29	7,243	7.52	
	*S. flavofasciata* - SFLA	169	56.8	0.57	27,828	12.17	
	*S. caribbeana* - SCAR	169	56.8	0.61	29,558	12.56	
	*S. pallens* - SPAL	168	57.6	1.41	66,005	13.58	
	*S. cancellata* - SCAN	170	57.6	1.08	51,109	13.02	
	*S. serialis cubense* - SSEC	172	58.8	0.98	61,898	21.81	
	*S. americana* - SAME	170	59.4	0.77	4,335	24.6	
	*S. damnifica* - SDAM	171	58.5	0.18	9,111	13.83	
	*S. ceratiola* - SCER	170	58.6	0.35	10,323	13.67	
	*S. rubiginosa -* SRUB	170	57.6	0.47	22,606	15.41	
	Average	170	57.5	0.7	29,002	14.8	22.5
	SD	1	1.8	0.4	23,116	4.9	
	CV (%)	0.6	3.2	58.0	79.7	33.2	

note.—SD, standard deviation; CV, coefficient of variation.

**Fig. 2.**
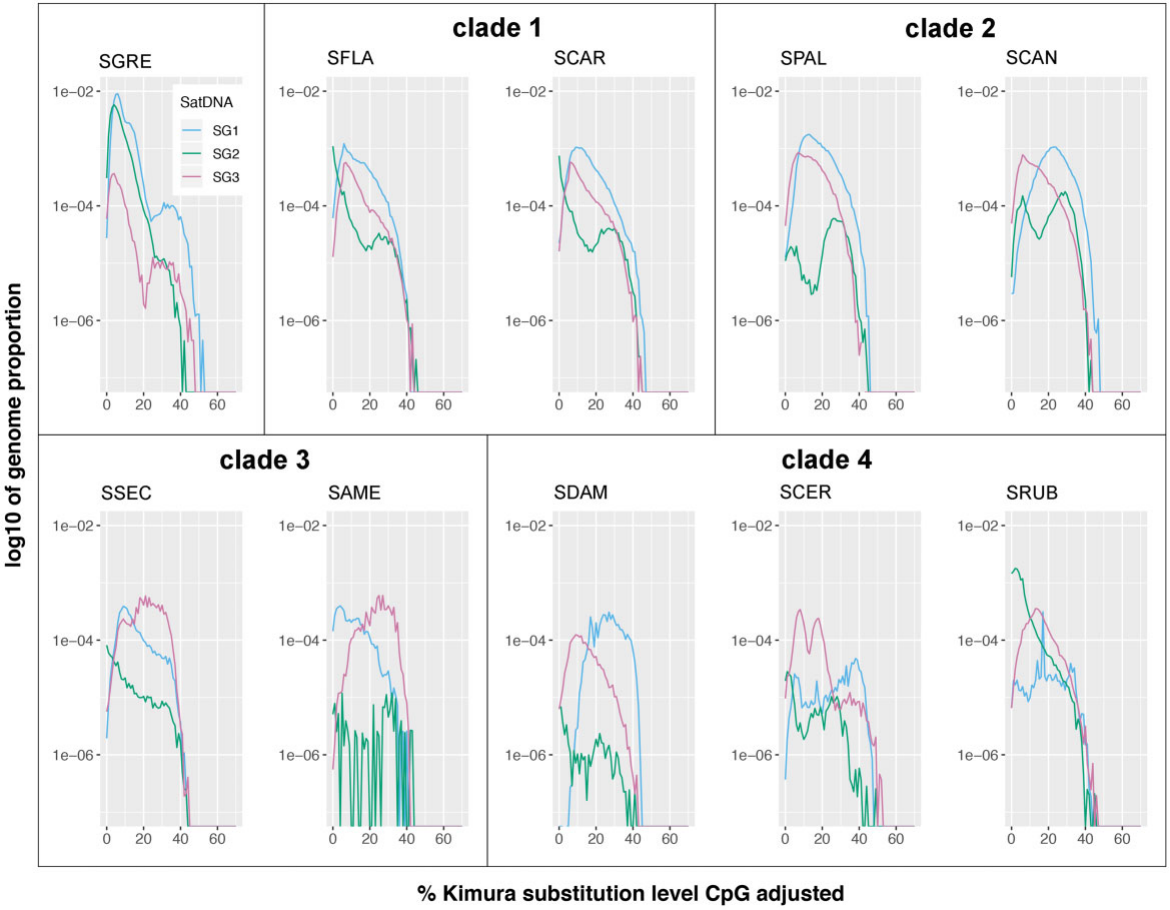
—SatDNA landscapes depicting the temporal accumulation of satDNAs in *Schistocerca* species. We created satDNA landscape plots depicting the repeat element divergence in K2P model distance to consensus on one axis and the satDNA abundance in logarithm scale on the other axis. Note we quantified repeats with a wide range of relative ages across the genomes.

We built repeat landscapes for SG1–SG3 in each species, which showed the abundance of the different genomic variants displaying different values of divergence. Assuming that satDNA sequence evolution mainly depends on point mutation (which increases sequence divergence) and homogenizing amplification (which decreases intraspecific divergence), it is logical to infer that the repeat landscape for a given satDNA displays temporal changes in abundance. Remarkably, figure 2 shows that repeat landscapes were more similar within clades rather than between clades, suggesting that evolutionary changes in satDNA closely reflect the evolutionary history of the species, as predicted by the library hypothesis (Fry and Salser 1977).

The analysis of *z*-score standardized values of abundance and divergence (zs-abun and zs-div) per species revealed interesting tendencies of satDNA evolution in these species. Supplementary figure 3, Supplementary Material online, shows high similarity between species within clades, in consistency with the repeat landscapes shown in figure 2. In addition, it shows that SG1 and SG2 are overabundant (i.e., zs-abun ≫ 0) in *S. gregaria* (with >2.5 standard deviations higher than the ten species average), in both cases with large decreases in divergence (zs-div ≪ 0). We infer that this combination of positive zs-abun and negative zs-div values indicates recent amplification of both satDNAs in this species, so that the decrease in intraspecific divergence (i.e., homogenization) caused by amplification has not yet been counteracted by the increase in divergence caused by point mutation, as the latter is expected to be proportional to the time lapse since the last amplification.

**Fig. 3.**
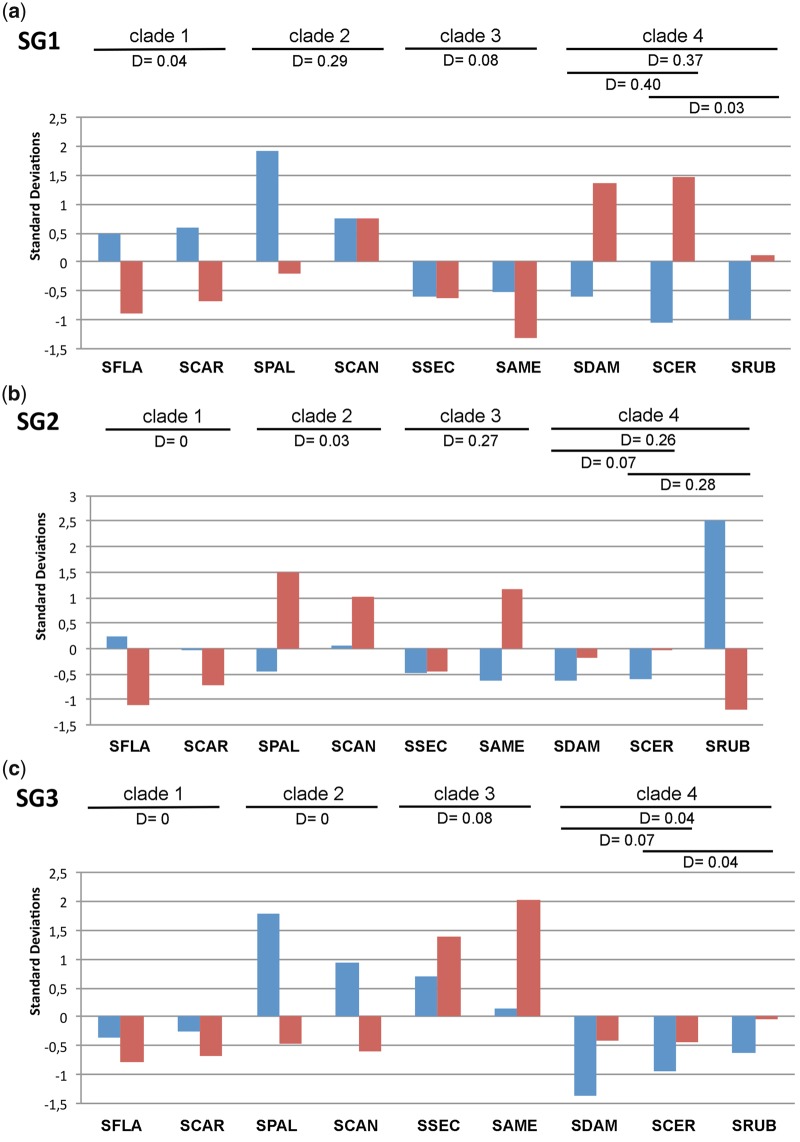
—*z*-Score standardized abundance (blue) and divergence (red) for SG1–SG3 in the nine American *Schistocerca* species. Pairwise distances (*D*), measured as *p* distance, are depicted for each phylogenetic clade.

The recent amplification of SG1 and SG2 in SGRE indicates that the library in this species has changed very much in respect to the ancestor library (i.e., that in the individuals which colonized America 7.9 Ma). Similar massive amplifications for a satDNA and an endogenous retrovirus in *Pan* and *Gorilla*, which were absent in *Homo* and *Pongo*, distorted the phylogenetic signal of genomic repeat abundance in the analysis of hominid phylogeny (Martín-Peciña et al. 2019). A way to solve this problem in *Schistocerca* is recalculating the zs-abun and zs-div values by discarding SGRE and assuming that the average for the nine American species represents the abundance probably present in the ancestral satDNA library of the American colonizers. A way to see the impact of this assumption is that it allowed detecting satDNA amplifications in some American species that had remained obscured by the huge amplifications in SGRE. For instance, the SG1 zs-abun values for SFLA and SCAR were negative when SGRE was included (supplementary fig. 3*a*, Supplementary Material online) but positive when it was excluded (fig. 3*a*). The new zs-abun values thus revealed SG1 amplification in clade 1 species prior to their separation, about 1.5 Ma (see also supplementary fig. 1, Supplementary Material online). These two species also showed highly similar abundance for SG3 (table 1), but it was lower than average (fig. 3*c*), thus pointing to an ancestral contraction of this satDNA. For SG2, however, these sister species showed abundance differences (fig. 3*b*), due to recent independent amplification in SFLA.

Following the same rationale, the two sister species included in clade 2 (SPAL and SCAN), which shared a common ancestor about 2 Ma (see supplementary fig. 1, Supplementary Material online), showed amplification for SG1 (which was larger and more recent in SPAL) and some contraction for SG2 (only in SPAL), whereas SG3 showed recent amplification in both species. Likewise, clade 3 species (SSEC and SAME) showed about the same patterns of change, with loss for SG1 and SG2 and gain for SG3. Finally, clade 4 species showed loss for all three satDNAs except in SRUB where a recent massive amplification for SG2 probably occurred in the B chromosome (fig. 3).

**Fig. 4.**
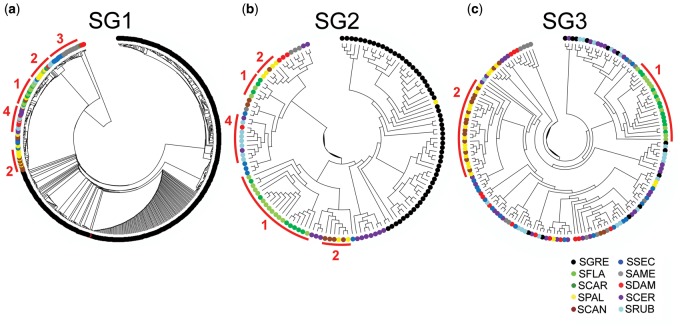
—ML tree showing the sequences relationship of the SG1, SG2, and SG3 assembled copies in *Schistocerca*. Note that in all cases the sequences from *S. gregaria* are in basal placement, which is in concordance with phylogenetic mitochondrial data that stated SGRE as the earliest divergent lineage. Occurrence of grouped sequences from more related species (belonging to the same clade) were observed as indicated by numbers 1–4. The nodal supports were calculated following 1,000 bootstrap replicates. The bootstrap support ≥ 90 are shown in supplementary figure 4, Supplementary Material online.

The ML trees built for SG1–SG3 (fig. 4) evidenced the existence of high sequence similarity between satDNA variants in different species, reflecting in the low branch supports. However, the SGRE sequences always appeared separated and basal mainly for SG1 and SG2, in consistency with its outgroup nature in the mitochondrial phylogenetic tree of the *Schistocerca* genus (Song et al. 2017). Although high similarity between satDNA variants of different species independent of their phylogenetic relationship was observed, interspecific groups from species belonging to the same clade were noticed, as for all clades of SG1; clades 1, 2, and 4 for SG2; and clades 1 and 2 for SG3 (fig. 4).

Sequence diversification between species, indicated by pairwise *p* distance (supplementary table 1, Supplementary Material online), was lower within rather than between clades for SG1 (Student *t *=* *2.09, df = 34, *P *=* *0.044) and SG3 (*t *=* *4.25, df = 34, *P *=* *0.00016), but not for SG2 (*t *=* *0.67, df = 34, *P *=* *0.51). This indicates that sequence changes in SG1 and SG3 were consistent with species phylogeny, but not those in SG2, perhaps due to the higher between species variation for abundance and intraspecific K2P divergence in the case of SG2. The substitution rates estimated from *p* distance were 2.1% (substitutions per million year) for SG1, 1.3% for SG2, and 1.5% for SG3. To test whether sequence changes are related with abundance changes, we compared within-clade distances with the zs-abun values (see fig. 3). In SG1, we found usually high within-clade consistency for abundance and distance for clades 1 and 3, and also between SCER and SRUB within clade 4. Remarkably, these two latter species have conserved very high sequence identity for SG1 (*D* = 0.03) in spite of the extreme contraction of this satDNA, as it represents 0.03% and 0.01% of the genome, respectively (table 1 and fig. 3*a*). However, clade 2 and clade 4 (SDAM in respect to SCER and SRUB) showed high sequence divergence (*D* = 0.29 and about 0.40, respectively) after a recent amplification in SPAL (clade 2) and contraction in clade 4 species (fig. 3*a*). On the other hand, SG2 showed scarce sequence diversification within clades 1 and 2 but it was much higher in clades 3 (due to higher K2P divergence in SAME than in SSEC) and 4 (due to the recent amplification in SRUB) (fig. 3*b*). Finally, in SG3, all distances were remarkably low (<0.1) indicating high sequence conservation for this satDNA, irrespectively of whether abundance figures indicated amplification (clades 2 and 3) or contraction (clades 1 and 4) (fig. 3*c*).

### Chromosomal Distribution of satDNAs

**Fig. 5.**
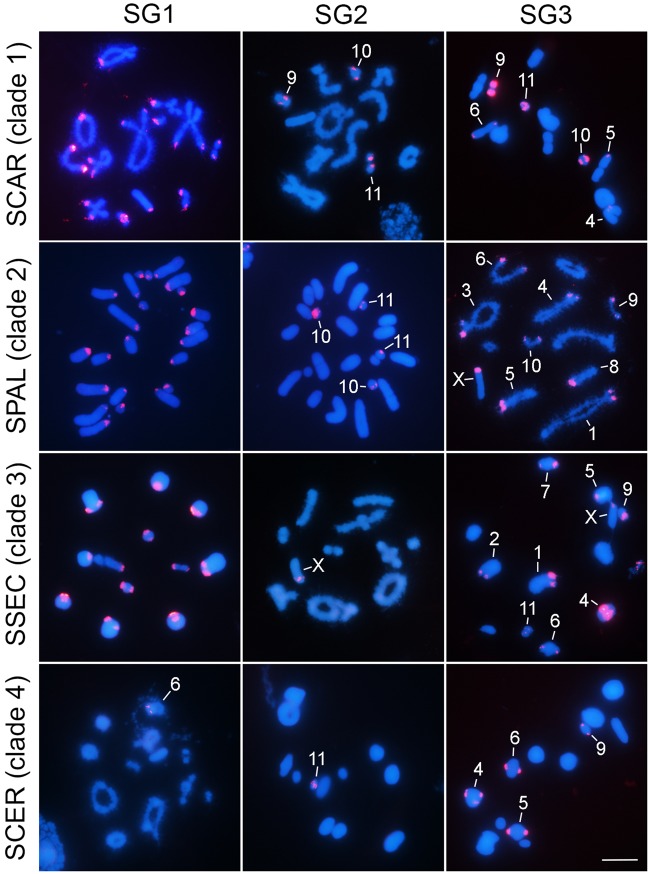
—FISH mapping (red signals) of SG1, SG2, and SG3 satDNAs on chromosomes counterstained with 4′,6-diamidine-2′-phenylindole (blue) in representatives of the four *Schistocerca* clades studied here. The FISH mapping confirms that C-positive heterochromatic blocks in *Schistocerca* species carry satDNAs, besides occurrence of some euchromatic blocks. FISH results for all species are presented in supplementary figures 5–7, Supplementary Material online. Chromosome names are indicated when signals show up in a few chromosomes.

**Fig. 6.**
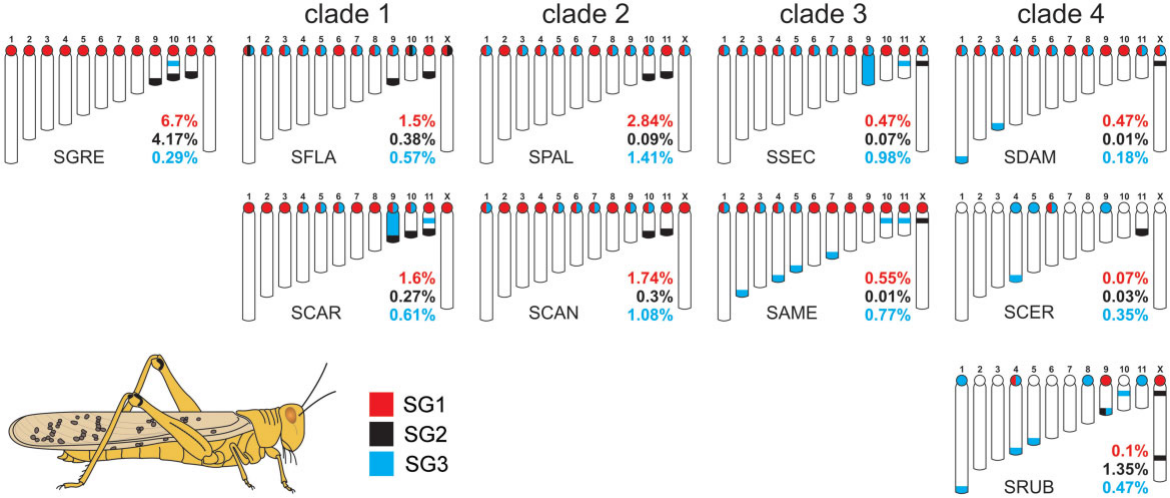
—Plots showing the satDNA location on chromosomes of *Schistocerca* species and their abundances. Data obtained from FISH mapping (chromosomal location) and RepeatMasker analysis (abundance). Note some concordance of distribution in distinct clades. Chromosomal location for SG3 was obtained from Camacho et al. (2015). SG1, red; SG2, black; SG3, blue.

FISH mapping of SG1–SG3 satDNAs showed their presence in the ten *Schistocerca* species (figs. 5 and 6 and supplementary figs. 5–7, Supplementary Material online). SG1 always showed pericentromeric location, whereas SG2 and SG3 were also found at interstitial and distal locations (see also supplementary table 2, Supplementary Material online).

When the FISH patterns were compared on a phylogenetic basis, SG1 showed 12 loci per haploid genome in all species except SCER and SRUB within clade 4, which only showed 1 and 3 loci, respectively (fig. 6). This was highly consistent with the extremely low abundance of this satDNA in these two species (see table 1 and fig. 3*a*).

In the case of SG2, clade 1 species displayed some differences for this satDNA location because SFLA showed pericentromeric loci on chromosomes 1, 10, and X, and distal bands on pairs 9 and 11, whereas, in SCAR, the bands were restrict to pairs 9–11 and all were distal (fig. 6 and supplementary table 2, Supplementary Material online). However, clade 2 species coincided in showing distal loci on autosomes 10 and 11, whereas clade 3 species coincided for an interstitial locus on the X chromosome (fig. 6 and supplementary table 2, Supplementary Material online). Finally, clade 4 species showed the highest differences, as they only coincided in the presence of an interstitial locus on the X chromosome of SDAM and SRUB, the remaining locations being distal and species specific (fig. 6 and supplementary table 2, Supplementary Material online).

On the other hand, the SG3 satDNA showed a complex pattern of coincidences and differences between species, with poor phylogenetic correspondence, except that distal FISH bands were observed only in species from clades 3 and 4 (fig. 6 and supplementary table 2, Supplementary Material online).

Spearman rank correlation analysis between satDNA abundance estimated bioinformatically (table 1) and the number of loci observed by FISH (supplementary table 2, Supplementary Material online) indicated positive correlation for SG1 (*ρ* = 0.70, *t* = 2.79, *P *=* *0.023) and SG2 (*ρ* = 0.87, *t* = 5.07, *P *=* *0.001), but not for SG3 (*ρ* = 0.10, *t* = 0.28, *P *=* *0.787). A possible explanation for this difference is that locus size appears to be more heterogeneous for SG3 (supplementary fig. 7, Supplementary Material online) rather than for SG1 and SG2 (supplementary figs. 5 and 6, Supplementary Material online).

A final comment merits the presence of satDNA on C-positive heterochromatic blocks. In general, we observed the presence of one or more satDNAs on pericentromeric regions, in coincidence with the pericentromeric C-bands. The exceptions were SCER and SRUB in which 8 and 6 chromosomes, respectively, lacked the three satDNAs on pericentromeric locations (fig. 6 and supplementary table 2, Supplementary Material online). In addition, the distal C-bands observed in SGRE (autosomes 9–11) contained the SG2 satDNA (fig. 1 and supplementary table 2, Supplementary Material online). However, our results clearly show the presence of satDNA outside any C-positive heterochromatic block, such as the SG2 loci at distal location on autosomes 9–11 in SCAR, autosomes 11 in SFLA, autosomes 10 and 11 in SPAL and SCAN, autosome 9 in SRUB, or autosome 11 in SCER. Furthermore, none of the interstitial loci for SG2 mapped on the X chromosome of SSEC, SAME, SDAM, and SRUB coincided with a heterochromatin block detectable by C-banding. Likewise, we observed that all interstitial and distal loci for SG3 on the autosomes S9–S11 and L1–M7 of several species (fig. 3) reside outside the heterochromatin defined by C-banding (fig. 1 and supplementary table 2, Supplementary Material online).

Finally, the presence of the three satDNA families on the B chromosome found in SRUB supports the hypothesis by Milani et al. (2018) claiming that the B chromosome arose from the S9 autosome, as this is the only A chromosome carrying the three satDNAs like the B chromosome (supplementary table 2, Supplementary Material online).

## Discussion

Our combined approach of low-coverage high-throughput sequencing and FISH mapping in ten grasshopper species belonging to the genus *Schistocerca* has revealed that the three satDNA families analyzed are conserved in the ten species, indicating that these satDNAs were part of the “library” present almost 8 Ma in the ancestor. During this time, the three satDNA families have displayed several amplifications, contractions, and point mutations across species, as evidenced by species differences in satDNA abundance and intraspecific K2P divergence, analyzed in combination with the number of FISH loci and the existing *Schistocerca* molecular phylogeny. First, repeat landscapes (see fig. 2) and *z*-score abundance and divergence values (fig. 3) showed high similarity between species within clades, indicating that in this specific case, evolutionary changes in satDNA abundance and divergence closely reflect species phylogeny. Second, changes at sequence level also reflected species phylogeny, because the ML trees built with satDNA sequence variants grouped together the sequences coming from the species sharing a same phylogenetic clade (see fig. 4).

It means that between species the differences in satDNA abundance and nucleotide sequence are proportional to the time because they shared their last common ancestor. In fact, the two pairs of sister species included in clades 1 and 2 showed higher similitude in abundance–divergence patterns rather than those in clades 3 and 4, in which phylogenetic relationships were more complex. Our results have shown that during the 7.9 Myr since the origin of the genus *Schistocerca*, estimated by Song et al. (2017), the satDNA library has experienced both gains and losses in abundance in different evolutionary lineages, the largest being noticed for SG1 and SG2 in the African species, SGRE. With the available information, it is impossible to know the precise figures for these satDNAs in the ancestor that colonized America, but it is likely that it might be close to the average found by us for the nine American species. Bearing this in mind, several evolutionary trends were observed for each of these three satDNAs. First, the high average K2P divergence values observed in all species (17.8% for SG1, 13.1% for SG2, and 14.8% for SG3) suggest that all three satDNAs are quite old in the genome of these grasshoppers. In fact, the rates of nucleotide substitution estimated here (2.1%, 1.5%, and 1.3%, respectively) are proportional to K2P values. These rates are slightly higher than those previously reported, for instance, in *Iberolacerta* lacertids (Rojo et al. 2015), sturgeons (Robles et al. 2004), and cetacean (Arnason et al. 1992).

Second, the SG1 family showed massive amplification in the African SGRE lineage reaching 6.7% of genome proportion. The other remarkable evolutionary trend for this satDNA was its contraction in clades 3 and 4, with extreme outcomes in SCER and SRUB in which it represented only 0.07% and 0.10% of the genome (see table 1). FISH mapping showed the presence of SG1 on pericentromeric regions of all chromosomes in all species with abundances close to 0.50% or higher, that is, all species except these two in which it was located only on the M6 chromosome pair (in SCER) or the M4, S9, and X (in SRUB). The *Schistocerca* phylogeny (see supplementary fig. 1, Supplementary Material online) indicates that this took place during the last 2 Myr. At least in SRUB, it is known that SG1 contraction has been paralleled by the presence of a more abundant nonhomologous satDNA, named SruSat02-170, on pericentromeric regions of all chromosomes (Milani et al. 2018). We also found this satDNA in the African SGRE genome (representing 0.428% of its genome), suggesting that it was also present in the ancestral *Schistocerca* that colonized America, like in other American species with variable abundance. It is well known that relaxed evolutionary constraints on satDNA lead to high rates of sequence turnover, even for those involved in centromeric function (Walsh 1987; Dover 2002; Ugarković and Plohl 2002; Garrido-Ramos 2017). The loss of the SG1 family in SCER and SRUB and its replacement by a different pericentromeric satDNA (SruSat02-170 in the latter species) thus illustrate the turnover principle of the satDNA library. Interestingly, SG1 in SCER and SRUB shows highly remarkable conservation (*D* = 0.03) in spite of large contraction (0.07% and 0.1% of genome proportion, respectively). Although this high sequence similarity might be due to chance, we cannot rule out the possibility that this satDNA has been recruited for some function in these species genomes, as suggested for other organisms (revised by Garrido-Ramos [2017]).

Third, the SG2 satDNA family experienced massive amplification in SGRE yielding large distal blocks on the S9–S11 chromosome pairs (see Camacho et al. 2015 and supplementary table 2, Supplementary Material online). This satDNA thus reached 4.17% of the genome in this species, whereas it only surpassed 1% in SRUB but presumably on a B chromosome (see above). Abundance thus ranged from 0.01% to 0.38% (average = 0.15%) in the eight other American species, indicating large contractions in SSEC (0.07%), SAME (0.01%), SDAM (0.01%), and SCER (0.03%) (see table 1), that is, the only species showing a single SG2 locus (see fig. 6 and supplementary table 2, Supplementary Material online). For SAME, interestingly sequences existing only in one chromosomal loci of X chromosome probably had undergone old amplification events (high K2P, that is not observed in the other three species with one SG2 locus) harboring now an excess of sequences differences between copies, which reflected as subgroups of nonhomogenized sequences.

The presence of loci on small chromosomes in clade 1 species, which is the second most basal in the American species, suggests that they were already present in the ancestral *Schistocerca*. In this case, we can infer that most distal loci on small chromosomes have been lost in clades 3 and 4 after their separation, about 4 Ma (see supplementary fig. 1, Supplementary Material online), from clade 2 species (which show distal loci on two small chromosomes). On the other hand, most species in clades 3 and 4 carried interstitial loci for SG2 on the X chromosome (see supplementary table 2, Supplementary Material online). This difference between the two groups of American species is also apparent for the zs-abun values (see fig. 3*b*), perhaps because loci on the small autosomes tend to be larger than those on the X chromosome (see supplementary fig. 6, Supplementary Material online). On the other hand, the absence of the X chromosome locus in SGRE and clade 1 species would be consistent with its ancestral absence, but its absence in clade 2 is more problematic. A way to explain it would be that it emerged after clade 1 separation (about 5 Ma) but was lost in clade 2 after its separation from clade 3. Alternatively, on the basis of satDNA dissemination hypothesis (Ruiz-Ruano et al. 2016), which claims that satDNAs are disseminated across genomes in the form of short arrays which may amplify on one chromosome site or another, the presence or absence of the loci on small autosomes, and X chromosome for SG2 might be the result of local amplifications or contractions. As the possible mechanisms for these events are mostly unknown, although many have been suggested (for a recent review, see Lower et al. [2018]), it is currently impossible to ascertain which of the two alternative explanations is more parsimonious.

Fourth, in the case of SG3, the main evolutionary changes were amplification in clades 2 and 3 and contraction on *S. gregaria* and clades 1 and 4. The present results show that there is no any correlation between abundances changes and loci number detected by FISH for SG3, perhaps due to locus-size heterogeneity. The SG3 repeat showed the highest variability in loci number and in chromosomal distribution and, as evidenced by FISH mapping, it was highly amplified in the orthologous chromosomes S9 in SCAR and SSEC across their whole length. Furthermore, interstitial loci for SG3 on one or two S-chromosomes were apparent in SGRE, SSEC, SCAR, SAME, and SRUB, presumably indicating its presence in the ancestral *Schistocerca* that colonized America and its loss in the five other species (see fig. 6). Finally, SG3 showed another trend for distal location on L and M chromosomes in a clade 3 species (SAME) and all clade 4 species.

Summing up, the analysis of sequence changes suggests that satDNA amplification usually tends to decrease intraspecific divergence (see SG1 and SG2 K2P values for SGRE, SG1 ones for clade 1 in table 1, or SG2 ones for SRUB). However, careful inspection at interspecific level (fig. 3) reveals that either sequence conservation or divergence was associated with both amplification or contraction for SG1 and SG2, but only sequence conservation for SG3 irrespectively of whether it had undergone amplification (clades 2 and 3) or contraction (clades 1 and 4) (table 2). This suggests that sequence evolution of a given satDNA is influenced by its history of amplifications and/or contractions in each species, with chance playing a major role in some cases (e.g., SG1 and SG2) and functional constrains possibly also acting in others (e.g., SG3, as it showed very low *D* values in all cases). This indicates that the evolution of satDNA abundance and sequence is mostly neutral but perhaps shaped by natural selection in some cases (Lower et al. 2018).

**Table 2:** SatDNA Changes in Sequence and Abundance in the Four Clades of American *Schistocerca* Species

satDNA	Result	Change in Clade No.	
		Amplification	Contraction
SG1	Conservation	1	3, 4
	Divergence	2	4
SG2	Conservation	1	2
	Divergence	4	3, 4
SG3	Conservation	2, 3	1, 4
	Divergence	—	—

Taken together, our results indicate that interspecies changes in satDNA abundance in *Schistocerca* show higher consistency with species phylogeny rather than those on chromosome satDNA location. This is especially apparent in the case of SG3 in which the level of local amplification seems to show higher heterogeneity between loci. Perhaps the nature of amplification mechanisms combined with the availability of short arrays acting as seeds for amplification (sensu Ruiz-Ruano et al. 2016) might help to explain the observed differences between SG1–SG2 and SG3 satDNA evolutionary pathways. Experimental and theoretical work indicated that de novo formation of satDNA repeats through unequal crossing over is relatively easy, and that, once formed, the arrays tend to persist (Southern 1970; Smith 1976), although they may also be lost (Charlesworth et al. 1986). However, Fry and Salser (1977) found results in rodents which were incompatible with the former models, for which reason they proposed the “library model,” by which the genomes of a group of species share a common collection of satDNAs in which interspecies differences are mostly quantitative. This includes the notion that a given satDNA may undergo differential amplifications or contractions among species, such as those reported here in *Schistocerca*. In contrast, in *Drosophila*, the immense majority of interspecific differences for simple satDNA were gains, with very few losses, and most lineage-specific gains occurred at terminal branches (Wei et al. 2018). The gains observed by us in *Schistocerca* also included some terminal branches, such as SG1 and SG2 in SGRE, SG1 in SPAL, or SG2 in SRUB, but others involved full phylogenetic clades by occurring in an ancestor species, such as SG1 in clade 1 species.

Our present results have revealed that K2P divergence was lower at intra- than inter-specific level for all three satDNAs analyzed here (see table 1) within nine *Schistocerca* species engaged in a rapid radiation throughout the American continent during the last 7.9 Myr (Song et al. 2017). This concerted pattern is consistent with the library model expectations (Fry and Salser 1977) as sequence diversification is boosted by reproductive isolation yielding species-specific satDNA sequence variants. Our results have allowed us to look at the evolution of a satDNA library from the early Pleistocene to the present, and the ML trees have shown high identity at species and clade levels. Given that our analysis only included three satDNA families, and because grasshoppers usually have tens of different satDNA families in their satellitomes (see Ruiz-Ruano et al. 2016, 2017, 2018), it is presumable that a comparative analysis of the full satellitomes in these ten species would shed interesting light on whether some satDNA families have appeared de novo in some of these genomes in such a short time, as predicted by Southern (1970), or else these species show coincident satellitomes, as predicted by the library model (Fry and Salser 1977).

## Supplementary Material

evaa018_Supplementary_Data

## Notes

Data deposition: This project has been deposited at GenBank under the accession MK948933–MK948959.
