# Supplementary material for: Eight Million Years of Satellite DNA Evolution in Grasshoppers of the Genus Schistocerca Illuminate the Ins and Outs of the Library Hypothesis
Source: Genome Biol Evol. 2020 Mar 17;12(3):88–102. doi: 10.1093/gbe/evaa018 (PMC7093836; doi:10.1093/gbe/evaa018)
Supplement: evaa018_Supplementary_Data [file evaa018_supplementary_data.zip › supp_table_1.docx]

**Supplementary Table 1.** Interspecific distance inferred by pairwise *p* distance between sat DNAs in *Schistocerca* species.

| SG1 |  |  |  |  |  |  |  |  |  |  |  |
| --- | --- | --- | --- | --- | --- | --- | --- | --- | --- | --- | --- |
|  |  | SGRE | SFLA | SCAR | SPAL | SCAN | SSER | SAME | SDAM | SCER | SRUB |
|  | SGRE | 0 |  |  |  |  |  |  |  |  |  |
|  | SFLA | 0,263 | 0 |  |  |  |  |  |  |  |  |
|  | SCAR | 0,249 | 0,04 | 0 |  |  |  |  |  |  |  |
|  | SPAL | 0,304 | 0,14 | 0,094 | 0 |  |  |  |  |  |  |
|  | SCAN | 0,312 | 0,253 | 0,214 | 0,288 | 0 |  |  |  |  |  |
|  | SSER | 0,433 | 0,339 | 0,325 | 0,388 | 0,423 | 0 |  |  |  |  |
|  | SAME | 0,5 | 0,406 | 0,393 | 0,448 | 0,46 | 0,082 | 0 |  |  |  |
|  | SDAM | 0,251 | 0,251 | 0,218 | 0,269 | 0,319 | 0,404 | 0,472 | 0 |  |  |
|  | SCER | 0,284 | 0,141 | 0,107 | 0,1 | 0,28 | 0,393 | 0,46 | 0,267 | 0 |  |
|  | SRUB | 0,365 | 0,164 | 0,135 | 0,099 | 0,314 | 0,409 | 0,463 | 0,301 | 0,148 | 0 |
| SG2 |  |  |  |  |  |  |  |  |  |  |  |
|  |  | SGRE | SFLA | SCAR | SPAL | SCAN | SSER | SAME | SDAM | SCER | SRUB |
|  | SGRE | 0 |  |  |  |  |  |  |  |  |  |
|  | SFLA | 0,169 | 0 |  |  |  |  |  |  |  |  |
|  | SCAR | 0,167 | 0 | 0 |  |  |  |  |  |  |  |
|  | SPAL | 0,151 | 0,094 | 0,091 | 0 |  |  |  |  |  |  |
|  | SCAN | 0,154 | 0,091 | 0,089 | 0,034 | 0 |  |  |  |  |  |
|  | SSER | 0,282 | 0,29 | 0,289 | 0,279 | 0,264 | 0 |  |  |  |  |
|  | SAME | 0,254 | 0,257 | 0,255 | 0,239 | 0,221 | 0,266 | 0 |  |  |  |
|  | SDAM | 0,251 | 0,253 | 0,249 | 0,25 | 0,232 | 0,238 | 0,149 | 0 |  |  |
|  | SCER | 0,128 | 0,153 | 0,151 | 0,142 | 0,148 | 0,269 | 0,244 | 0,232 | 0 |  |
|  | SRUB | 0,29 | 0,292 | 0,294 | 0,295 | 0,284 | 0,064 | 0,28 | 0,258 | 0,283 | 0 |
| SG3 |  |  |  |  |  |  |  |  |  |  |  |
|  |  | SGRE | SFLA | SCAR | SPAL | SCAN | SSER | SAME | SDAM | SCER | SRUB |
|  | SGRE | 0 |  |  |  |  |  |  |  |  |  |
|  | SFLA | 0,213 | 0 |  |  |  |  |  |  |  |  |
|  | SCAR | 0,213 | 0 | 0 |  |  |  |  |  |  |  |
|  | SPAL | 0,256 | 0,138 | 0,138 | 0 |  |  |  |  |  |  |
|  | SCAN | 0,253 | 0,136 | 0,136 | 0 | 0 |  |  |  |  |  |
|  | SSER | 0,218 | 0,118 | 0,118 | 0,06 | 0,059 | 0 |  |  |  |  |
|  | SAME | 0,265 | 0,166 | 0,166 | 0,119 | 0,118 | 0,076 | 0 |  |  |  |
|  | SDAM | 0,235 | 0,154 | 0,154 | 0,095 | 0,094 | 0,047 | 0,112 | 0 |  |  |
|  | SCER | 0,237 | 0,137 | 0,137 | 0,066 | 0,065 | 0,036 | 0,089 | 0,071 | 0 |  |
|  | SRUB | 0,218 | 0,112 | 0,112 | 0,06 | 0,059 | 0,018 | 0,082 | 0,041 | 0,041 | 0 |
