# Supplementary material for: Eight Million Years of Satellite DNA Evolution in Grasshoppers of the Genus Schistocerca Illuminate the Ins and Outs of the Library Hypothesis
Source: Genome Biol Evol. 2020 Mar 17;12(3):88–102. doi: 10.1093/gbe/evaa018 (PMC7093836; doi:10.1093/gbe/evaa018)
Supplement: evaa018_Supplementary_Data [file evaa018_supplementary_data.zip › supp_table_2.docx]

**Supplementary Table 3.** Chromosomal location and counting of the loci number of SG1, and SG2 and SG3 in *Schistocerca* species

| Species | SG1 chromosome location | | | | | | | | | | | | | | Total |
| --- | --- | --- | --- | --- | --- | --- | --- | --- | --- | --- | --- | --- | --- | --- | --- |
|  | L1 | L2 | L3 | M4 | M5 | M6 | M7 | M8 | S9 | S10 | S11 | X | B |  | |
| *S. gregaria* | p | p | p | p | p | p | p | p | p | p | p | p | no | 12 | |
| *S. flavofasciata* | p | p | p | p | p | p | p | p | p | p | p | p | no | 12 | |
| *S. caribbeana* | p | p | p | p | p | p | p | p | p | p | p | p | no | 12 | |
| *S. pallens* | p | p | p | p | p | p | p | p | p | p | p | p | no | 12 | |
| *S. cancellata* | p | p | p | p | p | p | p | p | p | p | p | p | no | 12 | |
| *S. serialis cubense* | p | p | p | p | p | p | p | p | p | p | p | p | no | 12 | |
| *S. americana* | p | p | p | p | p | p | p | p | p | p | p | p | no | 12 | |
| *S. damnifica* | p | p | p | p | p | p | p | p | p | p | p | p | no | 12 | |
| *S. ceratiola* |  |  |  |  |  | p |  |  |  |  |  |  | no | 01 | |
| *S. rubiginosa* |  |  |  | p |  |  |  |  | p |  |  | p | + | 03 | |
| Total | 08 | 08 | 08 | 09 | 08 | 09 | 08 | 08 | 09 | 08 | 08 | 09 |  | 100 | |
| Total (p) | 08 | 08 | 08 | 09 | 08 | 09 | 08 | 08 | 09 | 08 | 08 | 09 |  | 100 | |
| Total (i) |  |  |  |  |  |  |  |  |  |  |  |  |  |  | |
| Total (d) |  |  |  |  |  |  |  |  |  |  |  |  |  |  | |
|  | SG2 chromosome location | | | | | | | | | | | | | |  |
|  | L1 | L2 | L3 | M4 | M5 | M6 | M7 | M8 | S9 | S10 | S11 | X | B |  | |
| *S. gregaria* |  |  |  |  |  |  |  |  | d | d | d |  | no | 03 | |
| *S. flavofasciata* | p |  |  |  |  |  |  |  | d | p | d | p | no | 05 | |
| *S. caribbeana* |  |  |  |  |  |  |  |  | d | d | d |  | no | 03 | |
| *S. pallens* |  |  |  |  |  |  |  |  |  | d | d |  | no | 02 | |
| *S. cancellata* |  |  |  |  |  |  |  |  |  | d | d |  | no | 02 | |
| *S. serialis cubense* |  |  |  |  |  |  |  |  |  |  |  | i | no | 01 | |
| *S. americana* |  |  |  |  |  |  |  |  |  |  |  | i | no | 01 | |
| *S. damnifica* |  |  |  |  |  |  |  |  |  |  |  | i | no | 01 | |
| *S. ceratiola* |  |  |  |  |  |  |  |  |  |  | d |  | no | 01 | |
| *S. rubiginosa* |  |  |  |  |  |  |  |  | d |  |  | 2i | + | 03 | |
| Total | 01 |  |  |  |  |  |  |  | 04 | 05 | 06 | 06 |  | 22 | |
| Total (p) | 01 |  |  |  |  |  |  |  |  | 01 |  | 01 |  | 03 | |
| Total (i) |  |  |  |  |  |  |  |  |  |  |  | 05 |  | 05 | |
| Total (d) |  |  |  |  |  |  |  |  | 04 | 04 | 06 |  |  | 14 | |
|  | SG3 chromosome location | | | | | | | | | | | | | |  |
|  | L1 | L2 | L3 | M4 | M5 | M6 | M7 | M8 | S9 | S10 | S11 | X | B |  | |
| *S. gregaria* |  |  |  |  |  |  |  |  |  | i |  |  | no | 01 | |
| *S. flavofasciata* | p | p | p | p | p |  | p | p | p | p |  |  | no | 09 | |
| *S. caribbeana* |  |  |  | p | p | p |  |  | p,i* | p | i |  | no | 07 | |
| *S. pallens* | p |  | p | p | p | p |  | p | p | p |  | p | no | 09 | |
| *S. cancellata* | p |  |  |  | p | p | p |  | p | p |  |  | no | 06 | |
| *S. serialis cubense* | p | p |  | p | p | p | p |  | p,i* |  | i | p | no | 10 | |
| *S. americana* | p | d | p | p,d | p,d |  | d |  |  | i | i |  | no | 10 | |
| *S. damnifica* | p,d | p | p,d | p | p | p |  | p |  |  | p | p | no | 11 | |
| *S. ceratiola* |  |  |  | p,d | p | p |  |  | p |  |  |  | no | 05 | |
| *S. rubiginosa* | p,d |  |  | p,d | d |  |  | p | d | i | p |  | + | 09 | |
| Total | 09 | 04 | 05 | 11 | 10 | 06 | 04 | 04 | 09 | 07 | 05 | 03 |  | 77 | |
| Total (p) | 07 | 03 | 04 | 08 | 08 | 06 | 03 | 04 | 06 | 04 | 02 | 03 |  | 58 | |
| Total (i) |  |  |  |  |  |  |  |  | 02 | 03 | 03 |  |  | 08 | |
| Total (d) | 02 | 01 | 01 | 03 | 02 |  | 01 |  | 01 |  |  |  |  | 11 | |
