# Supplementary material for: Eight Million Years of Satellite DNA Evolution in Grasshoppers of the Genus Schistocerca Illuminate the Ins and Outs of the Library Hypothesis
Source: Genome Biol Evol. 2020 Mar 17;12(3):88–102. doi: 10.1093/gbe/evaa018 (PMC7093836; doi:10.1093/gbe/evaa018)
Supplement: evaa018_Supplementary_Data [file evaa018_supplementary_data.zip › Supplementary material legends.docx]

**Supplementary material**

**Supplementary Figures**

**Supplementary Figure 1. Phylogeny of *Schistocerca* genus with timescale divergence obtained by Song et al. (2017) with minor adaptation.** Species in purple were studied in this work and in red are indicated the four distinct clades considered here for our analysis.

**Supplementary Figure 2. C-banding revealing the heterochromatin location in meiotic and mitotic cells in *Schistocerca* species.** (a) SCAR, (b) SCAN, (c) SSEC, (d) SAME, (e) SDAM, (f) SCER. For other species the C-banding patterns were obtained from previous manuscripts and are represented in figure 1, SGRE (Camacho et al. 2015), SRUB (Milani et al. 2018), SPAL and SFLA (Souza and Melo 2007). Bar = 5μm.

**Supplementary Figure 3. Z-score standardized abundance (blue) and divergence (red) for SG1-SG3 in the ten *Schistocerca* species.**

**Supplementary Figure 4. Maximum likelihood tree showing the sequences relationship of the SG1, SG2 and SG3 assembled copies in *Schistocerca*.** The bootstrap support ≥ 90 are shown. These trees correspond to the trees shown in figure 4.

**Supplementary Figure 5. Fluorescent *in situ* hybridization (FISH) mapping of SG1 satDNA (red signals) on meiotic and mitotic chromosomes spreading counterstained with DAPI (blue) in *Schistocerca*.** (a) SFLA, (b) SCAR, (c) SPAL, (d) SCAN, (e) SSEC, (f) SAME, (g) SDAM, (h) SCER, (i) SRUB. Bar = 5μm.

**Supplementary Figure 6. Fluorescent *in situ* hybridization (FISH) mapping of SG2 satDNA (red signals) on meiotic and mitotic chromosomes spreading counterstained with DAPI (blue) in *Schistocerca*.** (a) SFLA, (b) SCAR, (c) SPAL, (d) SCAN, (e) SSEC, (f) SAME, (g) SDAM, (h) SCER, (i) SRUB. Bar = 5μm.

**Supplementary Figure 7. Fluorescent *in situ* hybridization (FISH) mapping of SG3 satDNA (red signals) on meiotic and mitotic chromosomes spreading counterstained with DAPI (blue) in *Schistocerca*.** (a) SFLA, (b) SCAR, (c) SPAL, (d) SCAN, (e) SSEC, (f) SAME, (g) SDAM, (h) SCER, (i) SRUB. Bar = 5μm.

**Supplementary Tables**

**Supplementary Table 1. Interspecific distance inferred by pairwise *p* distance between sat DNAs in *Schistocerca* species.**

**Supplementary Table 2. Chromosomal location and counting of the loci number of SG1, and SG2 and SG3 in *Schistocerca* species.** We counted the total number of proximal (p), interstitial (i) and distal (d) loci per chromosome and per genome haploid.
