# Supplementary figures and images for: Eight Million Years of Satellite DNA Evolution in Grasshoppers of the Genus Schistocerca Illuminate the Ins and Outs of the Library Hypothesis
Source: Genome Biol Evol. 2020 Mar 17;12(3):88–102. doi: 10.1093/gbe/evaa018 (PMC7093836; doi:10.1093/gbe/evaa018)

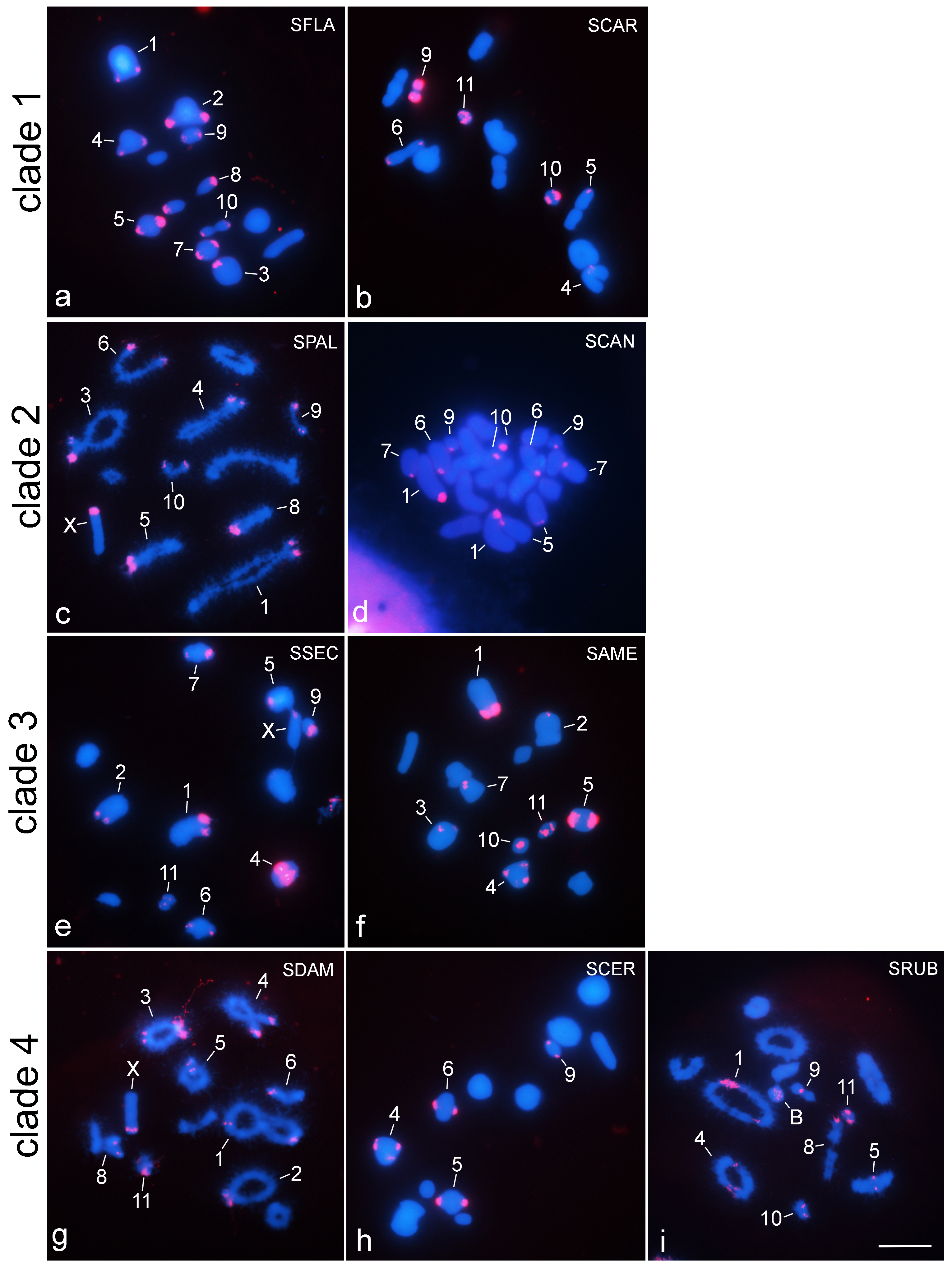

Supplement: evaa018_Supplementary_Data [file evaa018_supplementary_data.zip › supp_figure_7.jpg]

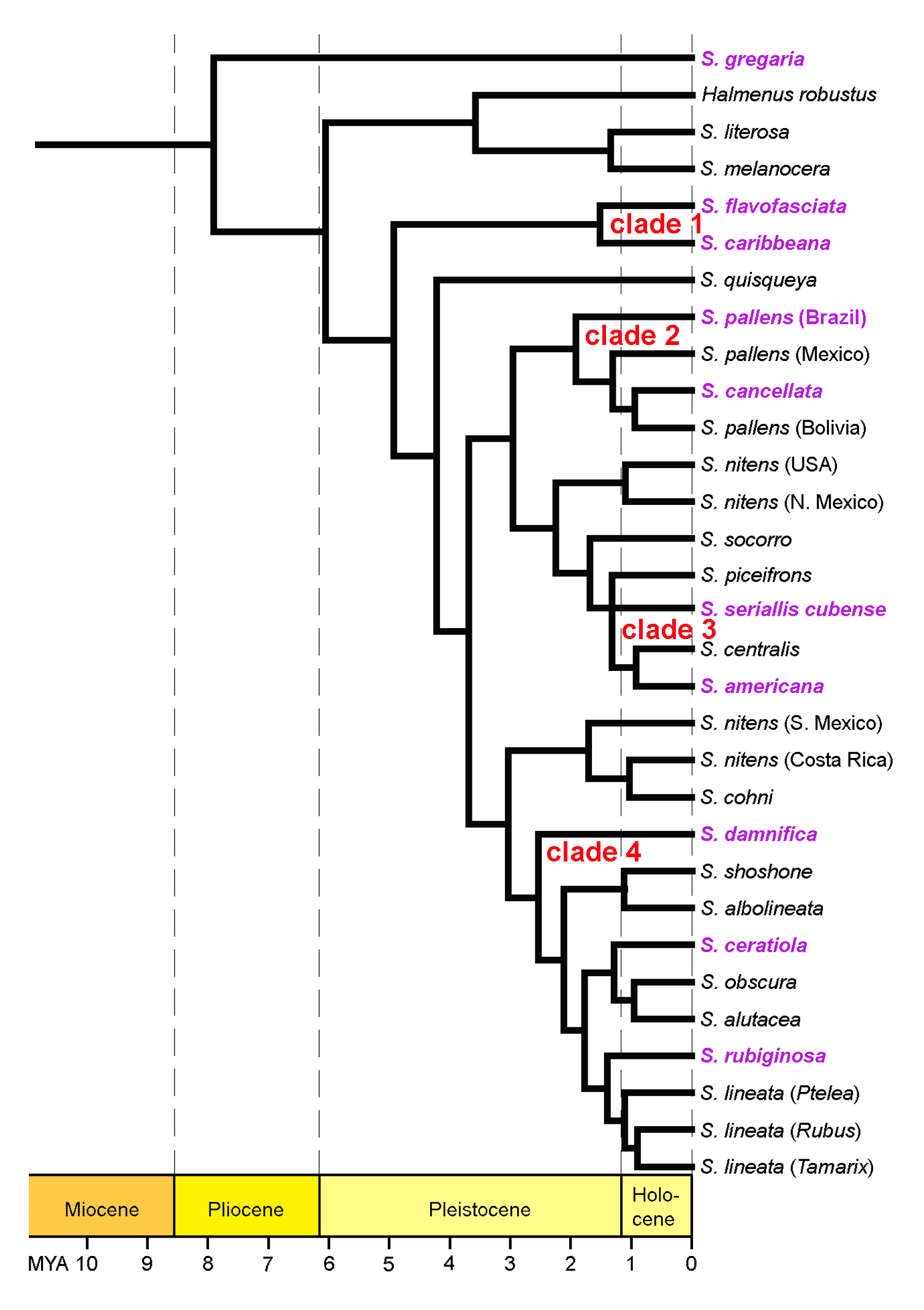

Supplement: evaa018_Supplementary_Data [file evaa018_supplementary_data.zip › supp_figure_1.jpg]

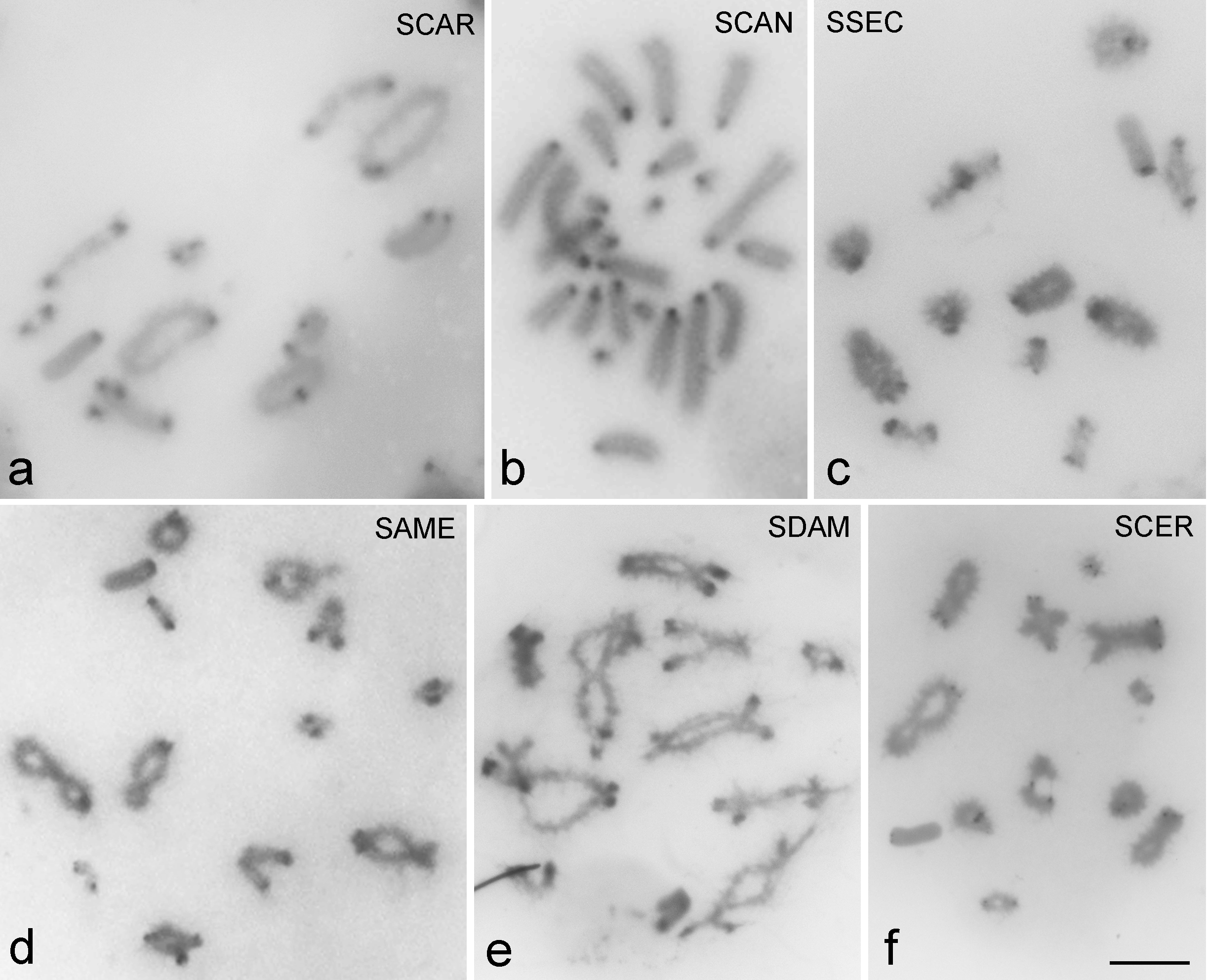

Supplement: evaa018_Supplementary_Data [file evaa018_supplementary_data.zip › supp_figure_2.jpg]

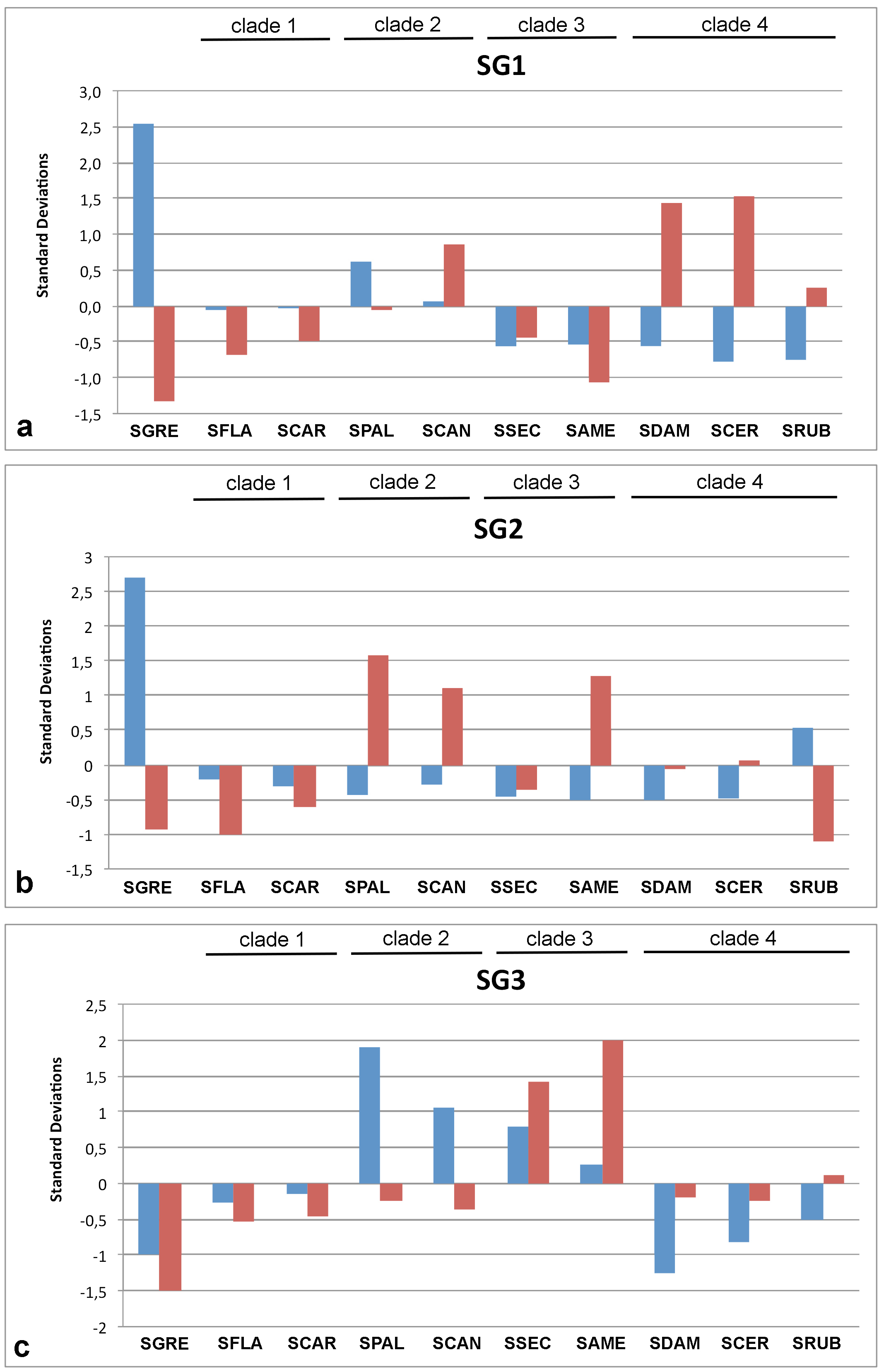

Supplement: evaa018_Supplementary_Data [file evaa018_supplementary_data.zip › supp_figure_3.jpg]

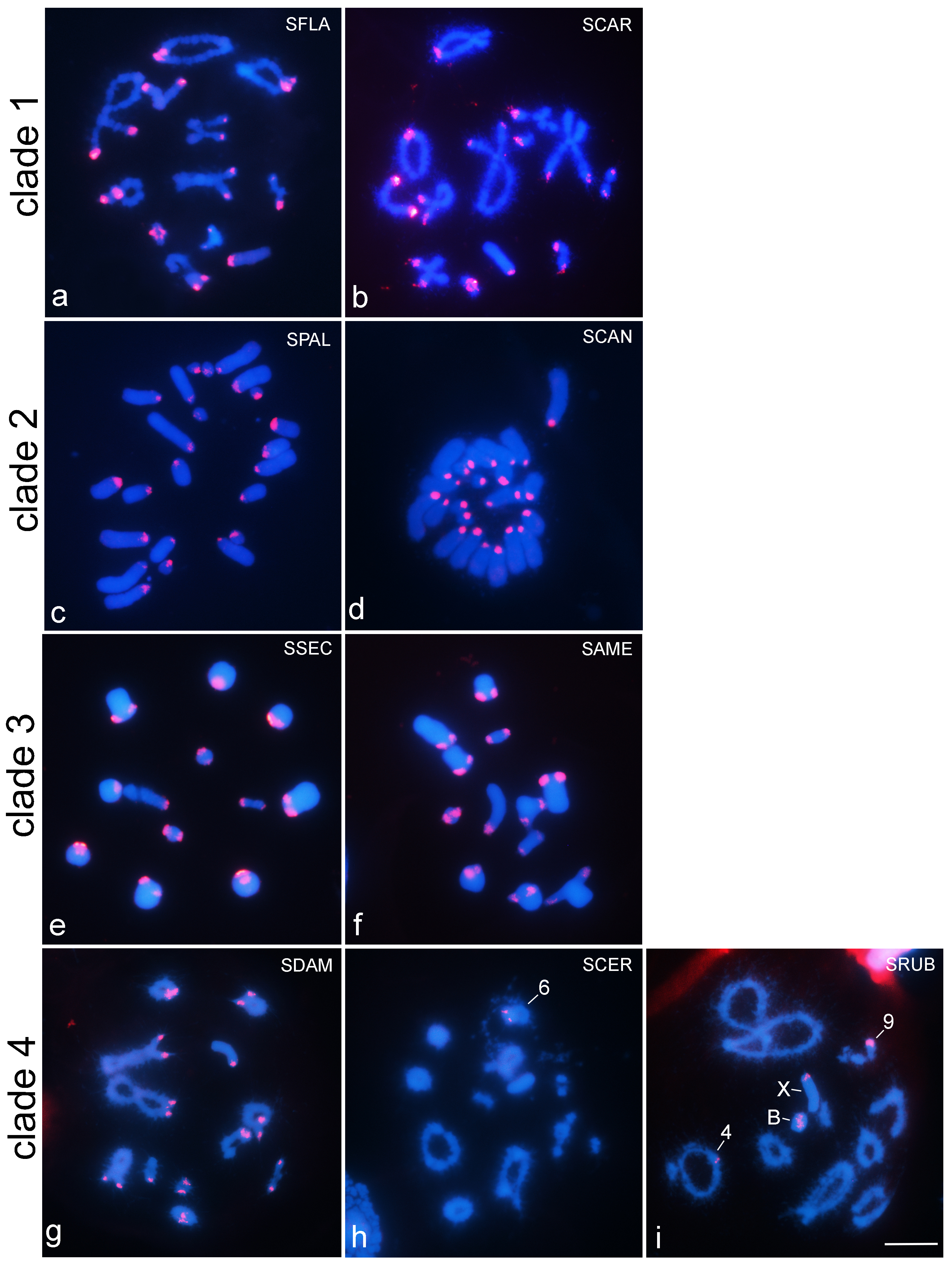

Supplement: evaa018_Supplementary_Data [file evaa018_supplementary_data.zip › supp_figure_5.jpg]

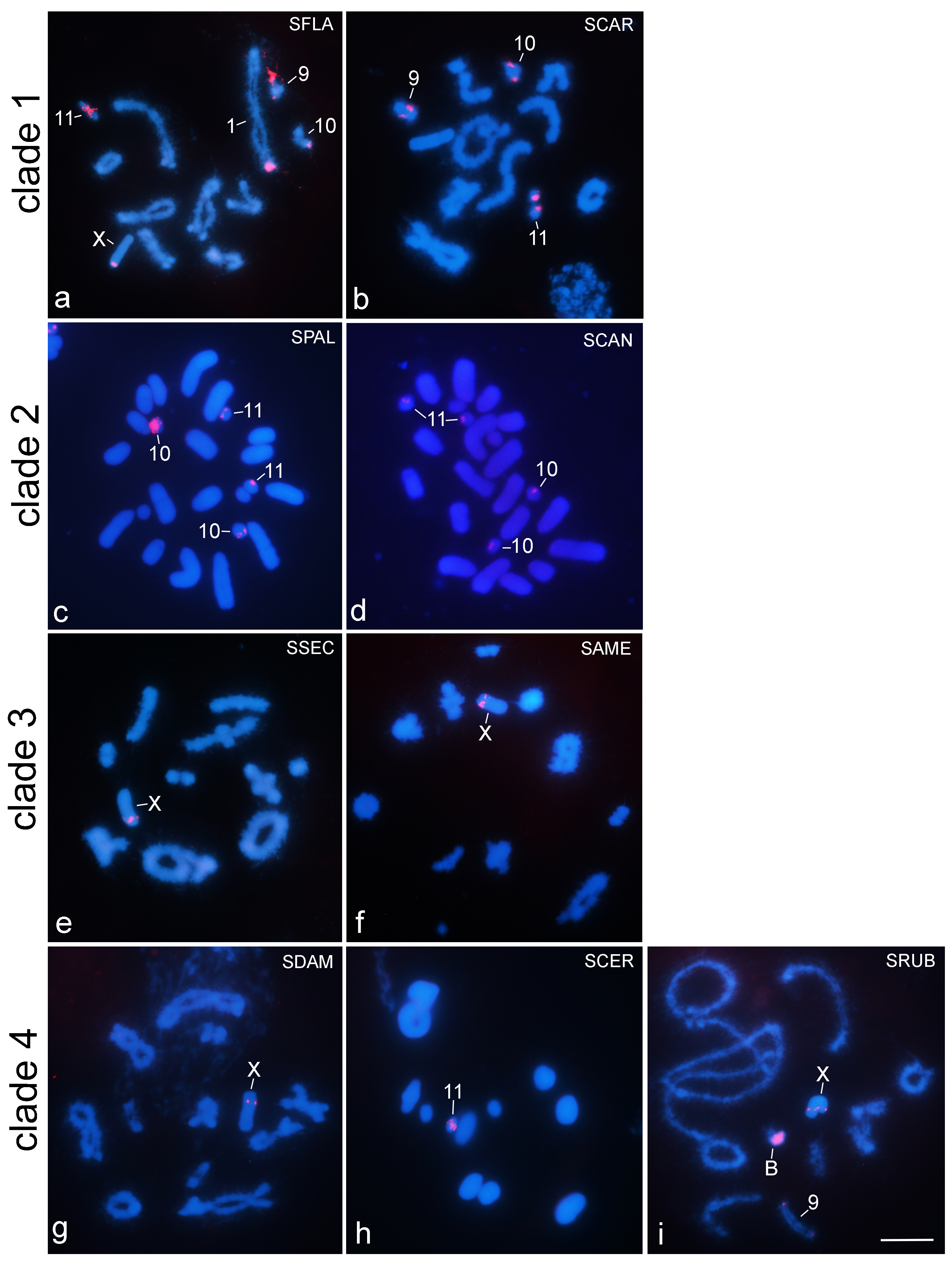

Supplement: evaa018_Supplementary_Data [file evaa018_supplementary_data.zip › supp_figure_6.jpg]
