# Supplementary material for: Eight Million Years of Satellite DNA Evolution in Grasshoppers of the Genus Schistocerca Illuminate the Ins and Outs of the Library Hypothesis
Source: Genome Biol Evol. 2020 Mar 17;12(3):88–102. doi: 10.1093/gbe/evaa018 (PMC7093836; doi:10.1093/gbe/evaa018)

(a) SG1

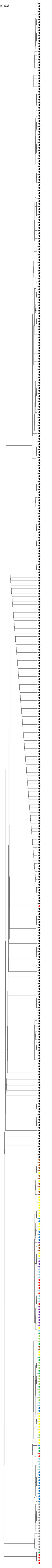

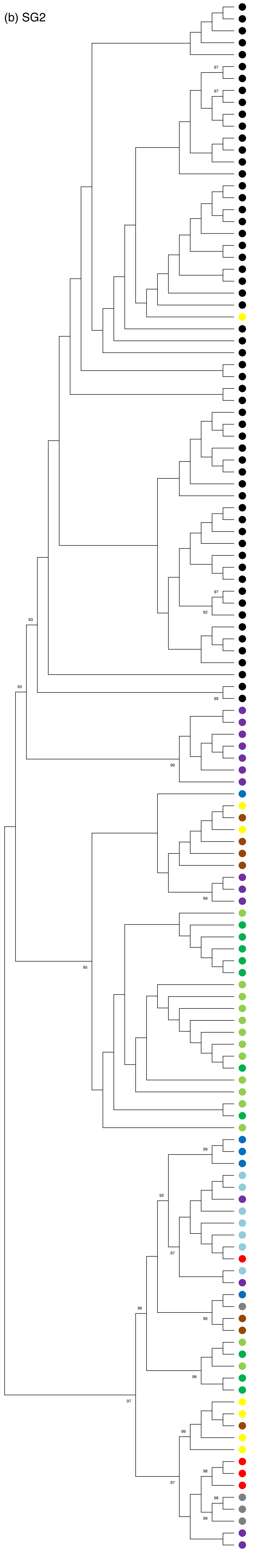
(b) SG2
97
97
93
93
97
92
99
99
99
95
99
92
97
96
96
98
97
99
97
98
98
99

(c) SG3

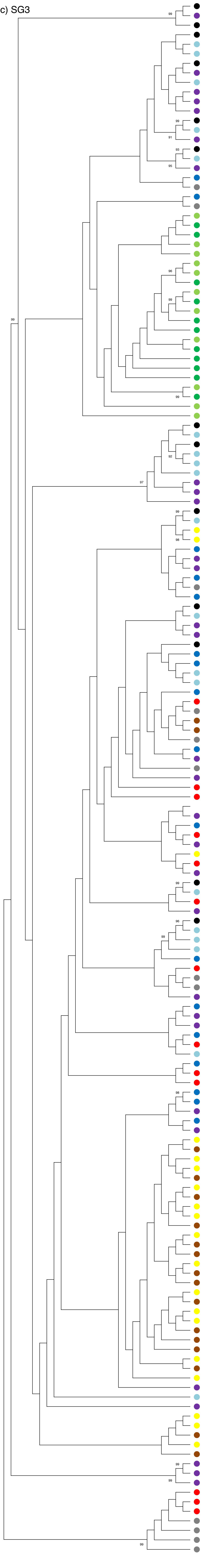

Supplement: evaa018_Supplementary_Data [file supp_figure_4.pdf › supp_figure_4.pdf]
